# Olfactory Responses of *Frankliniella occidentalis* and *Orius similis* to Volatiles from *Houttuynia cordata*: Implications for Thrip Management

**DOI:** 10.3390/plants14121855

**Published:** 2025-06-16

**Authors:** Guang Zeng, Shuo Lin, Feiyu Jiang, Changrong Zhang, Rongrong Yuan, Shuai Huang, Lijuan Wang, Yu Cao, Filippo Maggi, Giacinto Salvatore Germinara

**Affiliations:** 1 Department of Resources and Environment, Moutai Institute, Renhuai 564507, China; zengguang1992@126.com; 2 Institute of Plant Protection, Fujian Academy of Agricultural Sciences, Fuzhou 350000, China; 13075968592@163.com; 3 Guizhou Provincial Key Laboratory for Rare Animal and Economic Insect of the Mountainous Region, Guizhou Key Laboratory of Agricultural Biosecurity, Guiyang University, Guiyang 550005, China; 18585025745@163.com (F.J.); 18185682620@163.com (R.Y.); 19985269583@163.com (S.H.); wlj861015@163.com (L.W.); 4 Institute of Plant Protection, Guizhou Academy of Agricultural Sciences, Guiyang 550006, China; zhangchangrong2006@163.com; 5 Chemistry Interdisciplinary Project (ChIP) Research Center, School of Pharmacy, University of Camerino, 62032 Camerino, Italy; filippo.maggi@unicam.it; 6 Department of Agricultural Sciences, Food, Natural Resources and Engineering, University of Foggia, 71121 Foggia, Italy

**Keywords:** *Frankliniella occidentalis*, *Houttuynia cordata*, olfactory response, EAG response, volatiles, integrated pest management

## Abstract

Thrips can be attracted or repelled by volatiles from different host plant species. *Houttuynia cordata* is a common plant species with a strong, offensive smell, and few pests have been detected on this plant. Here, the olfactory responses of *Frankliniella occidentalis* to *H. cordata* volatiles were tested using electroantennography (EAG) and behavioral bioassays in different types of olfactometers, and the behavioral responses of *Orius similis*, a natural enemy of *F. occidentalis*, to the related main volatile compounds were also evaluated. Y-tube olfactometer bioassays showed that *F. occidentalis* performed negative responses to *H. cordata* volatiles. Decanal (47.21%), 1-decanol (11.02%), dodecanal (7.13%), *β*-myrcene (5.12%), and decanoyl acetaldehyde (3.76%) were the more abundant components in the *H. cordata* volatile profile in gas chromatography–mass spectrometry analysis. EAG recordings showed that the antennae of female thrips could perceive these five compounds at a wide range of concentrations. In six-arm olfactometer bioassays, *F. occidentalis* exhibited negative responses to decanal, dodecanal, and decanoyl acetaldehyde at various doses but performed positive responses to 1-decanol and *β*-myrcene at certain doses. Furthermore, decanal, dodecanal, and decanoyl acetaldehyde at all concentrations showed no significant influences on the behavioral responses of *O. similis*. According to the results above, *H. cordata* can be a repellent plant species to *F. occidentalis*, and decanal, dodecanal, and decanoyl acetaldehyde show great potential for development as repellents for the control of *F. occidentalis*. In short, our results suggest that an integrated pest management system combining *H. cordata*-derived biopesticides with releases of the predator *O. similis* could effectively control *F. occidentalis*.

## 1. Introduction

Western flower thrips, *Frankliniella occidentalis* (Pergande) (Thysanoptera: Thripidae), first arose as one of the world’s most significant invasive pests of agricultural and horticultural crops in the 1970s and 1980s [1]. *F. occidentalis* was originally described in 1895 in California, USA, and has since become an economically significant destructive pest with a geographic range that has rapidly expanded over the last 20 years. *F. occidentalis* causes direct damage through feeding and oviposition on leaves, flowers, and fruits, affecting yields and aesthetic appearance; it also causes indirect damage through the transmission of viruses [2,3].

As a phytophagous pest, *F. occidentalis* can attack over 250 crop species from over 60 families [4], yet its full host range is likely much broader, including vegetables, ornamentals, fruits, and cotton, among others [5]. In addition, *F. occidentalis* occurs on many uncultivated plants [6]. *F. occidentalis* has spread extensively through increased international trade and has become a dominant thrips species in many of the areas it has invaded [1,7,8]. Reliable estimates of economic losses are scarce and difficult to determine; however, given the high polyphagia of this pest as well as the plant pathogenic viruses it transmits, economic losses are likely very high [9,10]. Therefore, great attention has been paid to the effective control of this pest species.

For the control of *F. occidentalis*, reliance on a single management tactic, especially insecticides, has not been effective or sustainable [5,11]. Despite integrated pest management strategies being implemented, insecticides remain the cornerstone of many *F. occidentalis* management programs [5,12]. Such reliance on insecticides is unfortunate because *F. occidentalis* populations have a propensity for developing insecticide resistance, and resistance to several insecticide groups has been reported, including organophosphate, pyrethroid, and organochlorine pesticides [13,14,15,16]. Thus, novel strategies as alternatives to conventional insecticides for managing *F. occidentalis* are urgently needed.

In recent years, ethological control strategies have been considered more sustainable and environmentally friendly for pest management. However, most ethological control studies using semiochemicals have focused on larger insect pests, such as moths and beetles, with fewer studies on the numerous smaller insect pests, such as thrips [11,17]. Since the middle of the last century, the use of semiochemicals for pest control as a replacement for insecticides has been studied as a sustainable alternative [18]. Therefore, studies on thrips–host plant interactions are necessary for determining effective volatile organic compounds (VOCs) to implement practical *F. occidentalis* control methods [18]. Functional VOCs act as semiochemicals, which determine and/or modify the behavior of insects, through attraction or repulsion; in some cases, they are toxic to certain insect species [19,20].

*Houttuynia cordata* Thunb. (Piperales: Saururaceae), commonly known as fish mint, is a creeping aromatic medicinal herb with thin spreading rhizomes and a height of 30–60 cm [21]. It is an important traditional medicine in East and Southeast Asia, especially in China, Japan, and Thailand, where the plant is known as Kao-Tong or Plu Kao [22]. *H. cordata* can be commonly found in the wild and in cultivated fields in Guizhou Province, China; their fresh leaves and rhizomes are consumed as a vegetable, condiments, and spices. In particular, few phytophagous insects have been detected on this plant species, which may relate to the plant’s specific odor [22,23] that repels insects or is not preferred by them.

Therefore, in the present study, the behavioral and electrophysiological responses of *F. occidentalis* to the volatiles of fresh *H. cordata* leaves were investigated. Further, behavioral responses of *Orius similis* Zheng (Hemiptera: Anthocoridae), an important natural enemy of *F. occidentalis* [24], to the volatile compounds identified from *H. cordata* were also evaluated. Our study is based on the ecological regulation of “pest–host plant–natural enemy” for pest management. Taken together, these results can help narrow the gap between theoretical research and the practical application of behavioral regulation for pest control. In particular, this study provides useful information for the development of new attractants/repellents for the safe and sustainable control of *F. occidentalis* in agroforestry crops, in which *O. similis* can also be used to suppress *F. occidentalis* populations in combination with these semiochemicals.

## 2. Results

### 2.1. Behavioral Responses of Frankliniella occidentalis to Plant Volatiles

**Figure 1:**
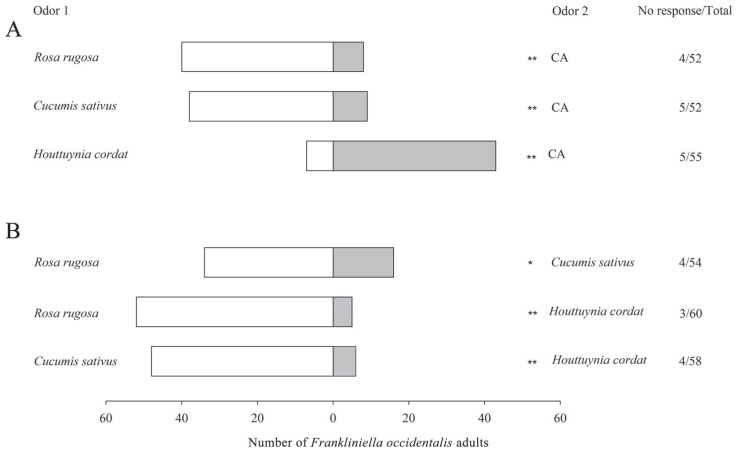
Behavioral responses of *Frankliniella occidentalis* to host plant odors in a Y-tube olfactometer. (**A**) host plant odors versus clean air; (**B**) host plant odors versus each other. CA: clean air. Asterisks indicate highly significant (** *p* < 0.01) and significant (* *p* < 0.05) differences in the selectivity of *F. occidentalis* between two paired odors by *χ*^2^ test.

In the Y-tube olfactometer bioassays, *F. occidentalis* showed significant responses when offered a choice between the odor of plant materials in one chamber and clean air in the other, responding positively to the volatiles of *Rosa rugosa* (*χ*^2^ = 21.33, *df* = 1, *p* < 0.001) and *Cucumis sativus* (*χ*^2^ = 17.89, *df* = 1, *p* < 0.001) but negatively to those of *H. cordata* (*χ*^2^ = 25.92, *df* = 1, *p* < 0.001) (Figure 1A).

Given a choice between pairs of these plant materials, *F. occidentalis* showed positive responses to the odors of *R. rugosa* paired with *C. sativus* (*χ*^2^ = 12.76, *df* = 1, *p* = 0.011) and *H. cordata* (*χ*^2^ = 38.75, *df* = 1, *p* < 0.001) and also showed positive responses to *C. sativus* paired with *H. cordata* (*χ*^2^ = 32.67, *df* = 1, *p* < 0.001) (Figure 1B).

### 2.2. Analysis of Houttuynia cordata Volatiles

Thirty-eight components were identified in the volatiles from *H. cordata* leaves (Table 1). The component with the highest relative content was decanal (47.21%), followed by 1-decanol (11.02%), dodecanal (7.13%), and *β*-myrcene (5.12%). There were no other components with a relative content exceeding 4%, but decanoyl acetaldehyde (3.76%), nonanal (3.50%), and undecanal (3.48%) also showed relatively higher contents compared with the other components in the volatile profile of *H. cordata* leaves.

### 2.3. Electroantennography Analyses

Two-way ANOVAs testing the effect of compound, and concentration on the EAG responses of *F. occidentalis* are shown in Table 2. The EAG responses of *F. occidentalis* were significantly affected by compound (*F* = 221.11, *p* < 0.001), concentration (*F* = 16.56, *p* < 0.001), and their interactions (*F* = 33.41, *p* < 0.001).

**Figure 2:**
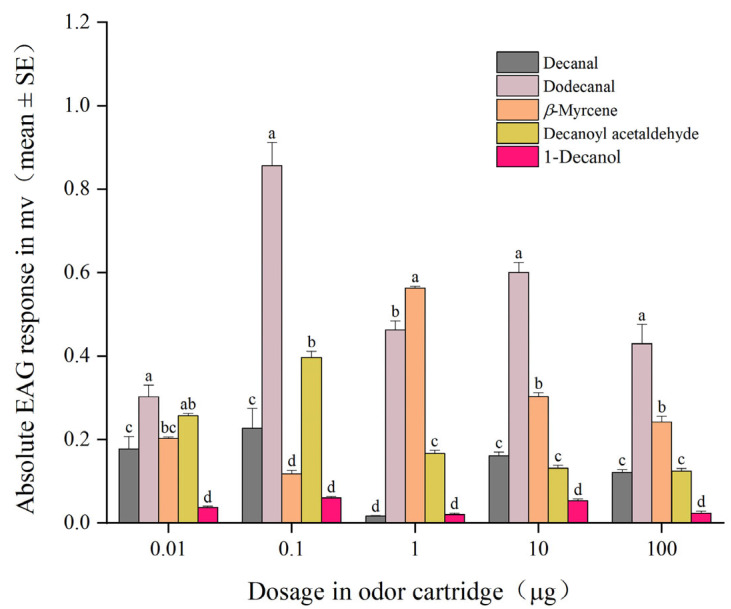
Electroantennography responses of *Frankliniella occidentalis* females to decanal, 1-decanol, dodecanal, *β*-myrcene, and decanoyl acetaldehyde. Mean values are shown. For each dose, different letters indicate significant differences at *p* < 0.05 (Tukey’s honest significant difference test).

Further, one-way ANOVA showed different responses of thrips to the five compounds at the 0.01 μg (*F*_4,20_ = 28.89, *p* < 0.001), 0.1 μg (*F*_4,20_ = 90.48, *p* < 0.001), 1 μg (*F*_4,20_ = 492.26, *p* < 0.001), 10 μg (*F*_4,20_ = 242.33, *p* < 0.001), and 100 μg (*F*_4,20_ = 48.84, *p* < 0.001) doses (Figure 2). The mean EAG response to dodecanal at 0.01 μg was significantly higher than those recorded for decanal, *β*-myrcene, and 1-decanol, but not significantly different from that to decanoyl acetaldehyde. At the 0.1, 10, and 100 μg doses, the mean EAG response to dodecanal was significantly higher than those to decanal, *β*-myrcene, and decanoyl acetaldehyde. At the 1 µg dose, the highest mean EAG response was recorded on stimulation with *β*-myrcene among the different stimuli tested.

### 2.4. Behavioral Responses of Frankliniella occidentalis to Houttuynia cordata Volatiles in a Six-Arm Olfactometer

**Figure 3:**
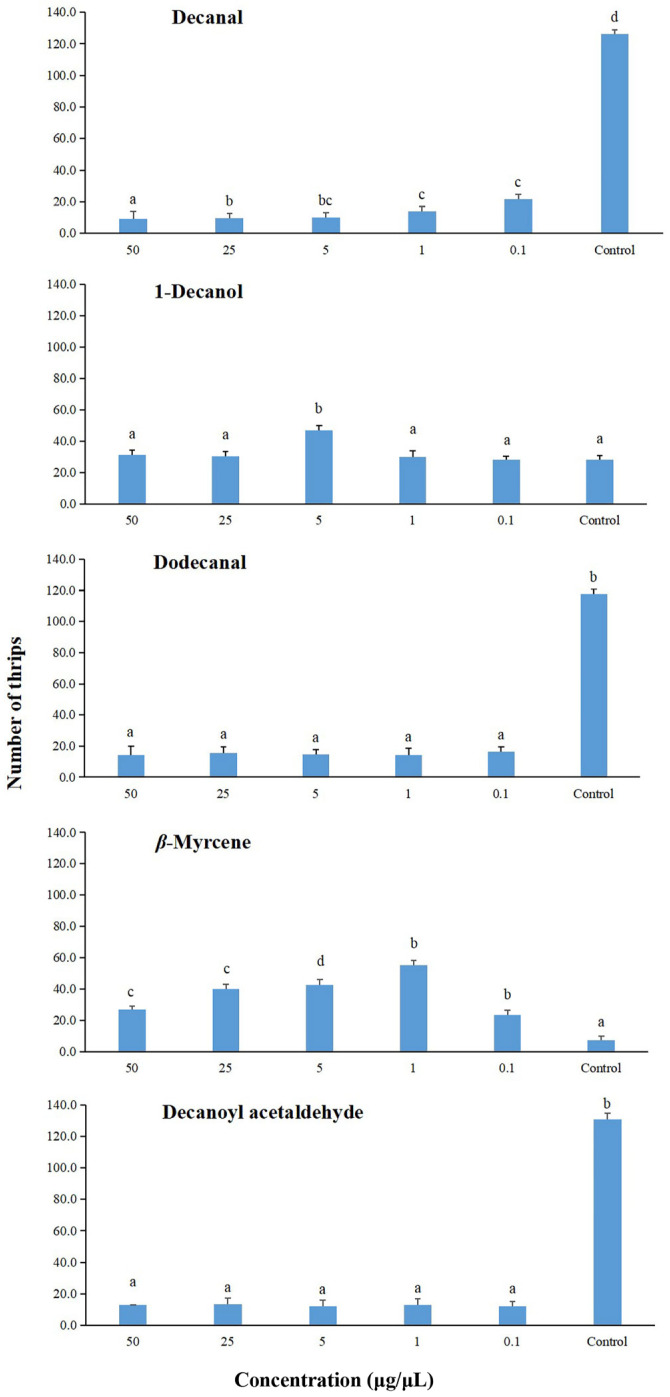
Olfactory responses of *Frankliniella occidentalis* to different compounds from the volatile profile of *H. cordata* in a six-arm olfactometer. The control was mineral oil. Each box plot represents the median and its range of dispersion (lower and upper quartiles and outliers). Above each box plot, different letters indicate significant differences (Wilcoxon test, *p* < 0.05).

In these bioassays, compared with the mineral oil control, *F. occidentalis* was repelled by decanal at all concentrations (Friedman test: *χ*^2^ = 22.56, *df* = 5, *p* < 0.001; Wilcoxon tests: *p* = 0.042–0.043), with the 50 µg/µl concentration eliciting a significantly higher repellent effect than the other concentrations (Figure 3). Similarly, compared with the mineral oil control, *F. occidentalis* was also repelled by dodecanal (Friedman test: *χ*^2^ = 14.05, *df* = 5, *p* < 0.015; Wilcoxon tests: *p* = 0.042–0.043) and decanoyl acetaldehyde (Friedman test: *χ*^2^ = 22.56, *df* = 5, *p* < 0.001; Wilcoxon tests: *p* = 0.042–0.043) at all concentrations, and there were no significant differences in repellent effect among the concentrations of each of these two compounds. *β*-Myrcene was attractant to *F. occidentalis* at various concentrations (Friedman test: *χ*^2^ = 24.00, *df* = 5, *p* < 0.001; Wilcoxon tests: *p* = 0.042–0.043), with the most attractant dose being 5 μg/μL. For 1-Decanol, thrips showed attraction only to the concentration of 5 μg/μL (Friedman test: *χ*^2^ = 14.45, *df* = 5, *p* < 0.001; Wilcoxon tests: *p* = 0.039–0.043).

### 2.5. Behavioral Responses of Predator Orius similis to compounds of Houttuynia cordata Volatiles in a Six-Arm Olfactometer

**Figure 4:**
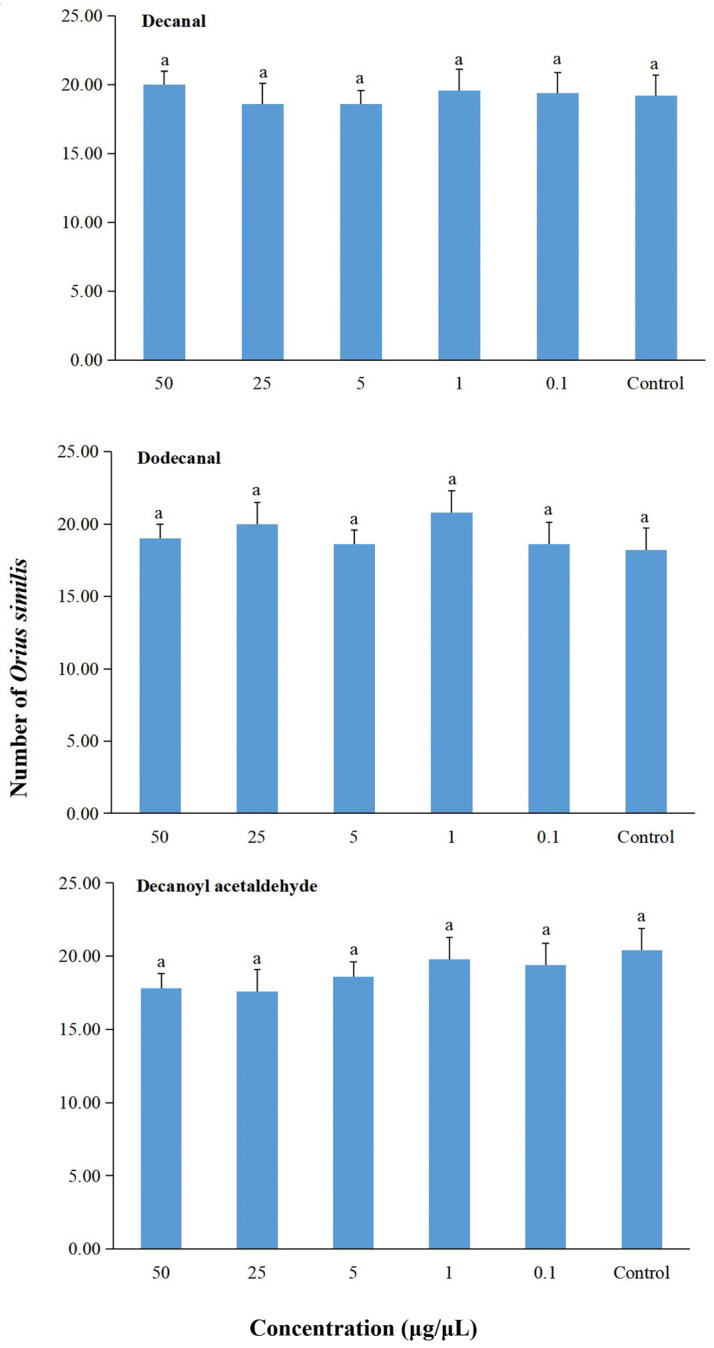
Behavioral responses of *Orius similis* to decanal, dodecanal, and decanoyl acetaldehyde in a six-arm olfactometer. The control was mineral oil. Each box plot represents the median and its range of dispersion (lower and upper quartiles and outliers). Above each box plot, the same letter indicates no significant differences (Friedman test, *p* > 0.05).

In the six-arm olfactometer bioassay, *O. similis* showed no attraction to decanal (Friedman test: *χ*^2^ = 3.44, *df* = 5, *p* = 0.632), dodecanal (Friedman test: *χ*^2^ = 3.33, *df* = 5, *p* < 0.649), or decanoyl acetaldehyde (Friedman test: *χ*^2^ = 6.39, *df* = 5, *p* = 0.270) at any concentration (Figure 4). There were no significant differences in the number of *O. similis* that entered into each arm for each concentration of these three compounds.

## 3. Discussion

It was reported that cucumber and rose flowers are the preferred host plants of *F. occidentalis* [25,26,27,28]. Here, when compared with cucumber and rose, *H. cordata* was not a preferred host plant, and it even repelled *F. occidentalis* in behavioral assays using the volatiles of *H. cordata* leaves. Usually, phytophagous insects rely on semiochemicals to search for suitable food, oviposition, and mating sites [29,30,31]. Therefore, the identification of behaviorally active compounds, either attractants or repellents, can provide a means for the monitoring and direct control of insect pests and can be used to improve integrated pest management systems.

Gas chromatography–mass spectrometry revealed 38 volatile compounds in *H. cordata*, among which decanal, 1-decanol, dodecanal, and *β*-myrcene were the most abundant. Further, EAG showed that these four main compounds were perceived by the peripheral olfactory systems of *F. occidentalis* females in a wide range of concentrations, with responses scaled in a dose-dependent manner. Although decanoyl acetaldehyde showed a relatively lower content among the volatile components of *H. cordata*, it is a characteristic component of the *H. cordata* odor [22,23]. Similarly to the above four main components, decanoyl acetaldehyde also elicited significant EAG responses in *F. occidentalis.*

In the six-arm olfactometer bioassays investigating the biological activity of these VOCs, *F. occidentalis* showed varying behavioral responses. *β*-Myrcene was attractive to *F. occidentalis* at various concentrations (0.1–50 μg/μL), and its most attractive concentration was 5 μg/μL. This compound has been reported as attractive at low concentrations to another thrip pollinator, *Cycadothrips chadwicki* (Thysanoptera: Aeolothripdae), and is also a repellant at high concentrations [32]. For 1-decanol, *F. occidentalis* showed no attraction except at the concentration of 5 μg/μL. Olfactory repellence was seen in *F. occidentalis* to the other three compounds (decanal, dodecanal, and decanoyl acetaldehyde) over the range of concentrations (0.1–50 μg/μL) used in this study. To a certain extent, this may explain why *F. occidentalis* exhibits negative responses to the odor of *H. cordata* leaves.

For thrips, the repellence of some plant odors can be attributed to single constituents. For example, *Megalurothrips sjostedti* (Thysanoptera: Thripidae) is repelled by volatiles from freshly cut lemongrass (*Cymbopogon citratus*) leaves, with one of the major compounds being the volatile monoterpenoid citral [33]. However, the influences of the ratios of plant volatiles on behavioral responses have also been highlighted for many phytophagous insects, including *F. occidentalis* [34,35,36,37]. Further, olfactory repellence in thrips has been demonstrated to be concentration-dependent, with some volatiles being effective only at specific concentrations and others over a range of concentrations [38]. Therefore, the present study used different concentrations of decanal, dodecanal, and decanoyl acetaldehyde individually, and it is possible that more compounds identified from the *H. cordata* volatiles are involved. The role of combinations of the VOCs and the ratio of their concentrations on the repellence or control of *F. occidentalis* should be further investigated in the field in future. In the laboratory, it is difficult to determine which VOCs repel thrips better, as most *F. occidentalis* chose clean air in the olfactometer bioassays.

There have been numerous studies on plant volatiles that are attractive or repellent to *F. occidentalis*. In these studies, *p*-anisaldehyde, benzaldehyde, ethyl nicotinate, nonanal, and other plant-produced semiochemicals have been reported to attract *F. occidentalis* in the laboratory, open field, and greenhouse [11,39,40,41,42,43], while 2-phenylethyl acetate, *β*-ionone, methyl salicylate, salicylaldehyde and other semiochemicals were found to repel *F. occidentalis* in the laboratory [27,38,44,45]. In addition, “insect pests–host plants–predators” systems regulated by semiochemicals can be useful in biological control strategies for the management of thrips [11,24,32]. For example, methyl isonicotinate can attract both *F. occidentalis* and their anthocorid predators (e.g., *Orius laevigatus* [Hemiptera: Anthocoridae]) [46]. Here, although *β*-myrcene was attractive to *F. occidentalis* at various concentrations, we focused on the repellent effects of VOCs on this thrip species for the purpose of pest control. Although decanal, dodecanal, and decanoyl acetaldehyde showed no attractiveness to *O. similis*, they were all repellent to *F. occidentalis.* Therefore, in the future, better control may be achieved by incorporating these volatile compounds as thrip-repellent biopesticides with biological control releases of *O. similis* in a multitactic integrated pest management system.

For interactions between thrips and host plants, prior to locating a potential host plant, thrips use plant volatiles as olfactory cues at a distance to avoid unsuitable hosts and at close range to avoid unsuitable plant parts. Therefore, based on the olfactory bioassays reported herein, *H. cordata* can be considered as an unsuitable host plant species for *F. occidentalis*, and decanal, dodecanal, and decanoyl acetaldehyde have the potential to be developed as repellents or deterrents for the control of *F. occidentalis.* Repellents cause insects to move away from an odor source and prevent adult insects from landing on a plant, further preventing contact between the insect and the stimulus [11,47,48]; further, odorants present on the plant surface or in the plant can act after alighting and may inhibit feeding or oviposition. It is difficult to discriminate repellents from deterrents, because the volatilization of compounds may give provide olfactory input even after contact with the plant [49,50]. In this manner, decanal, dodecanal, decanoyl acetaldehyde, and the other volatile chemicals identified herein should be further evaluated for their influence on the feeding, oviposition, and other behaviors of *F. occidentalis* during interactions with host plants. This will help in better understanding the physicochemical characteristics of these chemicals (e.g., used as repellents or deterrents) and making full use of them for the management of thrips as pest species. It has been reported that repellent volatiles may be involved in the resistance of chrysanthemum and cowpea cultivars to *F. occidentalis* [33,51], and these plants also show promise for developing cultivars with resistance to *F. occidentalis*.

## 4. Materials and Methods

### 4.1. Insects and Plants

Mixed populations of *F. occidentalis* collected from various host plant species in the Nanming District, Guiyang, Guizhou Province, China, were used to establish a laboratory colony [25]. The independent colony was continuously reared for more than five generations on bean pods of *Phaseolus vulgaris* L. (Fabales: Leguminosae) in plastic containers [25,26]. The containers were kept in a climate-controlled room at 26 ± 1 °C and 65 ± 5% relative humidity with a 14:10 h light/dark photoperiod.

*H. cordata* seedlings were collected from the wild in Guiyang, Guizhou Province, China, and planted in greenhouses in the nursery of Guiyang University (Guiyang, China); two common vegetable/flower host plant species of *F. occidentalis*, cucumber (*C. sativus* L. var. Qianyou No. 1) and rose (*R. rugosa* Thunb. var. Carola), were also grown in the nursery. These plants were cultivated without the application of pesticides. Rose flowers at anthesis with intact petals were collected for olfactory tests. Leaves of *H. cordata* and cucumber were used for experiments when the plants had eight true leaves.

### 4.2. Behavioral Responses of Frankliniella occidentalis to Plant Volatiles in a Y-Tube Olfactometer

The olfactory responses of *F. occidentalis* were tested in a Y-tube olfactometer using the method described in our previous study [25]. We made two types of comparisons: (1) each plant versus clean air and (2) all plants versus each other. Based on the results of previous studies, cucumber (*C. sativus* L. var. Qianyou No. 1) and rose (*R. rugosa* Thunb. var. Carola), were used in these comparisons [25,52]. The airflow rate was 250 mL/min. All bioassays were conducted between 08:00 and 18:00 in a room at 25 ± 1 °C, 65 ± 5% relative humidity, and 1000 lux illumination. For each comparison, 50–60 females that were 2–3 days old were tested individually, and there was 5 min for each thrip to make a choice or not [25]. Thrips were starved for 6 h before the bioassay, and the plant material (20.0 g) was replaced after every 10 tested individuals.

### 4.3. Gas Chromatography–Mass Spectrometry Analysis

Plant volatiles were collected and analyzed as described by Cao et al. [52]. Plant material (0.3 g) was kept in a glass bottle (200 mL) for 2 h before the volatiles emitted were captured using a solid-phase microextraction fiber (a ~50/30 µm DVB/CAR/PDMS StableFlex fiber). The volatiles were extracted for 40 min at 80 °C before the fiber head was quickly removed. The collected volatiles were analyzed using gas chromatography–mass spectrometry (HP6890/5975C; Agilent Technologies, Santa Clara, CA, USA). To identify compounds, we compared the mass spectra of compounds with those in databases (Nist 2005 and Wiley 275), and their constituents were confirmed through co-injection with authentic standards. The volatiles from the leaves of *H. cordata* were collected and analyzed.

### 4.4. Electroantennograms

The antennal sensitivity of *F. occidentalis* females (2–3 days old) to increasing concentrations of the five test compounds was evaluated using electroantennography (EAG) with a technique described by Abdullah et al. [53] and detailed in our previous study [34]. The antennae of *F. occidentalis* females were excised at the groove between antennal segments 7 and 8 (most distal to the head) to achieve better contact with electrodes. The antenna was placed between the head end of a reference electrode and the top of a recording electrode, with the aid of a micromanipulator. The EAG equipment (IDAC-2; Syntech GmbH, Kirchzarten, Germany) consisted of a data acquisition collector, an AC/DC amplifier, a stimulus flow controller, a single-ended probe, and a micromanipulator. To ensure electrical continuity between the antennal preparation and the EAG apparatus, the recording and reference electrodes were silver-coated wires in pulled glass micropipettes containing 0.5 M KCl conductive saline solution. For each test compound, 10 μL of different mineral oil solutions, providing doses of 0.01, 0.1, 1, 10, and 100 μg, was adsorbed onto a piece of filter paper (1 cm^2^, Whatman No. 1) inserted into a Pasteur pipette, which was used as an odor cartridge [34]. Mineral oil was used as the control in each experiment. Each dose of the four compounds was tested on five different antennae from different female thrips; each antenna sample was stimulated by each dose of each compound in the following order: control, sample, control. The average of the two controls was used for analysis. The EAG responses of thrips to the compounds were calculated as

Relative EAG value = T − CK,

where T is the absolute EAG value for the antennae samples and CK is the absolute EAG value for the control. For the five tested compounds, decanal (chemical purity ≥ 97%), dodecanal (chemical purity ≥ 95%), and *β*-myrcene (chemical purity ≥ 90%) were purchased from Shanghai Macklin Biochemical Co., Ltd., Shanghai, China; 1-decanol (chemical purity ≥ 97%) was purchased from Dr. A. Maisch GmbH (Baden-Württemberg, Germany); decanoyl acetaldehyde (chemical purity ≥ 95%) was purchased from National Institutes for Food and Drug Control, Beijing, China.

### 4.5. Behavioral Responses of Frankliniella occidentalis to Houttuynia cordata Volatiles in a Six-Arm Olfactometer

As decanal, 1-decanol, dodecanal, *β*-myrcene, and decanoyl acetaldehyde were the five compounds with the highest relative abundances in the volatile profile of *H. cordata*, behavioral responses of *F. occidentalis* to different doses of each of these compounds were further assessed in a six-arm olfactometer [54,55]. The six-arm olfactometer consisted of a central chamber (12 cm internal diameter) with six arms, each of which was connected to a glass tube (angles between pairs of tubes were 60°) that projected outwards at an equal distance. Each arm was connected with Teflon tubing to a glass vessel, which was used as the odor chamber that contained a test (10 µL of each compound solution at 0.1, 1, 5, 25, and 50 μg/μL) or control stimulus (10 μL mineral oil). The airflow was set at 200 mL/min to drive the odor towards the thrips. Female *F. occidentalis* (2–3 days old, starved for 6 h) were introduced into the olfactometer in groups of 200 individuals. The thrips were counted and considered as having made a choice for a particular odor source within 30 min, any thrips that entered into one of these arms were considered to make a choice [54,55,56]. The bioassays were replicated five times and carried out between 09:00 and 17:00 at room temperature (25 ± 2 °C).

### 4.6. Behavioral Responses of Orius similis to Houttuynia cordata Volatiles in a Six-Arm Olfactometer

To evaluate potential influences on the behavioral response of predators of *F. occidentalis*, the olfactory responses of *O. similis* to decanal, dodecanal, and decanoyl acetaldehyde at various concentrations mentioned above were also tested. Similar to the process for the six-arm olfactometer bioassay described above (see Section 4.5), *O. similis* (2–3 days old, starved for 8 h) were introduced into the olfactometer in groups of 120 individuals (1:1 male/female ratio). The airflow was set at 200 mL/min to drive the odor towards the *O. similis*. The bioassays were replicated five times, and each bioassay was accomplished within 30 min.

### 4.7. Statistical Analyses

All statistical analyses were performed using SPSS 20.0 for Windows (SPSS Inc., Chicago, IL, USA). The null hypothesis that *F. occidentalis* adults showed no preference for either Y-tube arm (a response equal to 50:50) was tested using a chi-squared goodness-of-fit test. The number of thrips and *O. similis* found in the different arms of the six-arm olfactometers were subjected to Friedman two-way ANOVA by ranks. In the case of significance (*p* < 0.05), the Wilcoxon signed ranks test was used for the separation of means. Two-way ANOVA was carried out to examine the effects of compound, concentration, and their interactions on the EAG responses of thrips. If ANOVA indicated significant treatment effects, one-way ANOVA followed by Tukey’s HSD test (*p* < 0.05) was used to test the separate means. Before ANOVA, data were submitted to the Shapiro–Wilk test to verify the normal distribution of data and to Levene’s test to assess the homogeneity of variances.

## 5. Conclusions

This study demonstrated that *F. occidentalis* showed negative olfactory responses to the volatiles of *H. cordata* leaves, which were closely related to the components of *H. cordata* volatiles. Further, decanal, dodecanal, and decanoyl acetaldehyde, identified from the volatile profile of *H. cordata*, had repellent effects on *F. occidentalis* but exhibited neither attraction nor repellence to *O. similis*, the natural predator of *F. occidentalis*. Therefore, new control measures for *F. occidentalis* can be considered by incorporating these volatile compounds (repellent effects) with the release of *O. similis* (preying on thrips).

## Figures and Tables

**Table 1:** Volatile components of *Houttuynia cordata* leaves.

Number	Compound	Molecular Formula	Molecular Weight	Content (%)
1	3-Hexenal	C_6_H_10_O	98	0.61
2	3-Hexen-1-ol, (E)-	C_6_H_12_O	100	2.15
3	2-Hexenal, (E)-	C_6_H_10_O	98	0.79
4	1-Hexanol	C_6_H_14_O	102	0.09
5	Nonane	C_9_H_20_	128	0.31
6	2,4-Hexadienal, (E,E)-	C_6_H_8_O	96	0.05
7	*α*-Thujene	C_10_H_16_	136	0.04
8	*α*-Pinene	C_10_H_16_	136	0.29
9	Camphene	C_10_H_16_	136	0.05
10	4-Oxohex-2-enal	C_6_H_8_O_2_	112	0.08
11	Sabinene	C_10_H_16_	136	1.57
12	*β*-Pinene	C_10_H_16_	136	0.41
13	*β*-Myrcene	C_10_H_16_	136	5.12
14	Octanal	C_8_H_16_O	128	0.06
15	*α*-Terpinene	C_10_H_16_	136	0.07
16	Limonene	C_10_H_16_	136	0.11
17	(*Z*)-*β*-Ocimene	C_10_H_16_	136	0.06
18	(*E*)-*β*-Ocimene	C_10_H_16_	136	1.69
19	*γ*-Terpinene	C_10_H_16_	136	0.11
20	Undecane	C_11_H_24_	156	0.06
21	Nonanal	C_9_H_18_O	142	3.50
22	1-Nonanol	C_9_H_20_O	144	1.32
23	Decanal	C_10_H_20_O	156	47.21
24	1-Decanol	C_10_H_22_O	158	11.02
25	l-Bornyl acetate	C_12_H_20_O_2_	196	0.12
26	2-Undecanone	C_11_H_22_O	170	1.65
27	Undecanal	C_11_H_22_O	170	3.48
28	n-Decanoic acid	C_10_H_20_O_2_	172	0.12
29	Geranyl acetate	C_12_H_20_O_2_	196	0.78
30	Dodecanal	C_12_H_24_O	184	7.13
31	Decanoyl acetaldehyde	C_12_H_22_O_2_	198	3.76
32	(*E*)-Caryophyllene	C_15_H_24_	204	0.86
33	*α*-Humulene	C_15_H_24_	204	0.08
34	(*E*)-*β*-Farnesene	C_15_H_24_	204	0.67
35	Bicyclogermacrene	C_15_H_24_	204	0.49
36	(*E*, *E*)-*α*-Farnesene	C_15_H_24_	204	0.15
37	Tetradecanal	C_14_H_28_O	212	0.47
38	Hexadecanal	C_16_H_32_O	240	0.07

**Table 2:** Two-way ANOVAs testing for the effects of compound and concentrations on the electroantennography responses of *Frankliniella occidentalis*.

Parameter	Source	*df*	MS	*F*	*p*
Relative EAG value (mv)	Compound	4	0.613	221.11	<0.001
	Concentration	4	0.046	16.56	<0.001
	Compound × Concentration	16	0.093	33.41	<0.001
	Error	80	0.003		

## Data Availability

The original contributions presented in this study are included in the article. Further inquiries can be directed to the corresponding author.
